# Supplementary material for: Knowledge, attitude and practice toward childhood immunization among mothers in Lebanon
Source: PLoS One. 2025 May 8;20(5):e0322205. doi: 10.1371/journal.pone.0322205 (PMC12061142; doi:10.1371/journal.pone.0322205)
Supplement: S1 Fig — (PDF) [file pone.0322205.s001.pdf]

QUESTIONNAIRE (ENGLISH VERSION) WITH PARTICIPANT'S AGREEMENT

In the following page, there is a set of questions that represents knowledge, attitude and practice of Lebanese mothers toward childhood immunization (have children aged between zero to five years).

Please read each statement carefully taking into consideration your clinical condition.

We are interested to learn your response for each statement.

Sociodemographic Characteristics of the Mothers

• Mother age

☐ 18 to 25 ☐ 25 to 31 ☐ 31 to 35 ☐ 35 or older

• Mother's educational level

☐uneducated

☐ Literate

☐ Primary school

☐ Secondary school

☐ High school

☐ Bachelor or higher

• Job status

☐Student ☐Employee ☐Housewife

• Monthly income

☐ Enough ☐ Partly enough ☐ Not enough

• Number of children

☐ One ☐ Two to three ☐ More than three

• Age of children

☐ 0 to 6 months ☐ 6 to 12 months

☐ 12 to 24 months ☐ 2 to 5 years

• Are you smoker (hookah included)

☐ Yes ☐ No

For each statement please put ☒ depending on your comprehension.

Mother’s knowledge:

• Is vaccination important for children from the first day of birth?

☐ agree ☐ uncertain ☐ disagree

• Does vaccination reduce death and disability?

☐ agree ☐ uncertain ☐ disagree

• Can vaccination keep children healthy?

☐ agree ☐ uncertain ☐ disagree

• Can childhood vaccinations control contagious diseases that affect children (measles, diphtheria, tetanus, pertussis and hepatitis B)?

☐ agree ☐ uncertain ☐ disagree

• Can a child be given more than one vaccine in a time?

☐ agree ☐ uncertain ☐ disagree

• Is there side effect from vaccines?

☐ agree ☐ uncertain ☐ disagree

If you agree, mention side effect happened with your child?

• Is there a link between vaccination and autism?

☐ agree ☐ uncertain ☐ disagree

• Does even a healthy child need vaccinations?

☐ agree ☐ uncertain ☐ disagree

• What is the source of your information about vaccinations?

☐ Primary healthcare center

☐ Friends and relatives

☐ Ministry of health

☐ Website

☐ Study

☐ Search

• Do you know how to boost your child’s immune system other than vaccination? ☐ agree ☐ uncertain ☐ disagree

If yes mention how?

Mother’s attitude:

• Do you think vaccinations are beneficial?

☐ agree ☐ uncertain ☐ disagree

• Do you feel that it is safe to have your child vaccinated?

☐ agree ☐ uncertain ☐ disagree

• Do you support the compulsory vaccination programs designed by the Ministry of Health?

☐ agree ☐ uncertain ☐ disagree

• Do you think nature immunity better than vaccination?

☐ agree ☐ uncertain ☐ disagree

• Do you advise your relatives and family to vaccinate their children?

☐ agree ☐ uncertain ☐ disagree

• Do you think that immunization check list is more beneficial to be written and recorded?

☐ agree ☐ uncertain ☐ disagree

• Do you think that quality of vaccine services differs between dispensary and private clinic?

☐ agree ☐ uncertain ☐ disagree

• Do you think that healthy life style increase child's immunity during childhood?

☐ agree ☐ uncertain ☐ disagree

• Do you think breastfeeding is essential to maintain immunity?

☐ agree ☐ uncertain ☐ disagree

Mother's practice:

• Has your child received the mandatory childhood vaccines?

☐ Yes ☐ No

• Have your children received optional vaccines? (influenza, Rota virus)?

☐ Yes ☐ No

If no, why?

☐ cost ☐ not important ☐ other

• Do you follow the compulsory vaccination programs listed in the vaccination schedule?

☐ Yes ☐ No

• Do you look for other vaccines available to your child?

☐ Yes ☐ No

• Do you use pain relievers to relieve swelling and pain after having your child vaccinated?

☐ Yes ☐ No

• Where did your child get his vaccination?

☐ dispensary ☐ private clinic ☐ others

• Who pay for the vaccine administration fee?

☐ ministry of health ☐ private insurance

☐ out of pocket ☐ other

• Do you follow a healthy lifestyle for your child?

☐ Yes ☐ No

• Did you follow breastfeeding?

☐ Yes ☐ No

If yes, mention duration of breastfeeding?

……………………………………………………………………………………………..

• Was the high cost of vaccines ever an obstacle for your child to get his vaccination?

☐ Yes ☐ No

• Was unavailability of vaccines during the crisis ever an obstacle for your child to get his vaccination?

☐ Yes ☐ No

Participant’s Agreement:

I have read the information provided above. I have asked all the questions I have at this time. I voluntarily agree to participate in this research study.

Signature: ____

Date: _________________

THANK YOU SO MUCH IN ADVANCE
