# Supplementary figures and images for: Knowledge, attitude and practice toward childhood immunization among mothers in Lebanon
Source: PLoS One. 2025 May 8;20(5):e0322205. doi: 10.1371/journal.pone.0322205 (PMC12061142; doi:10.1371/journal.pone.0322205)

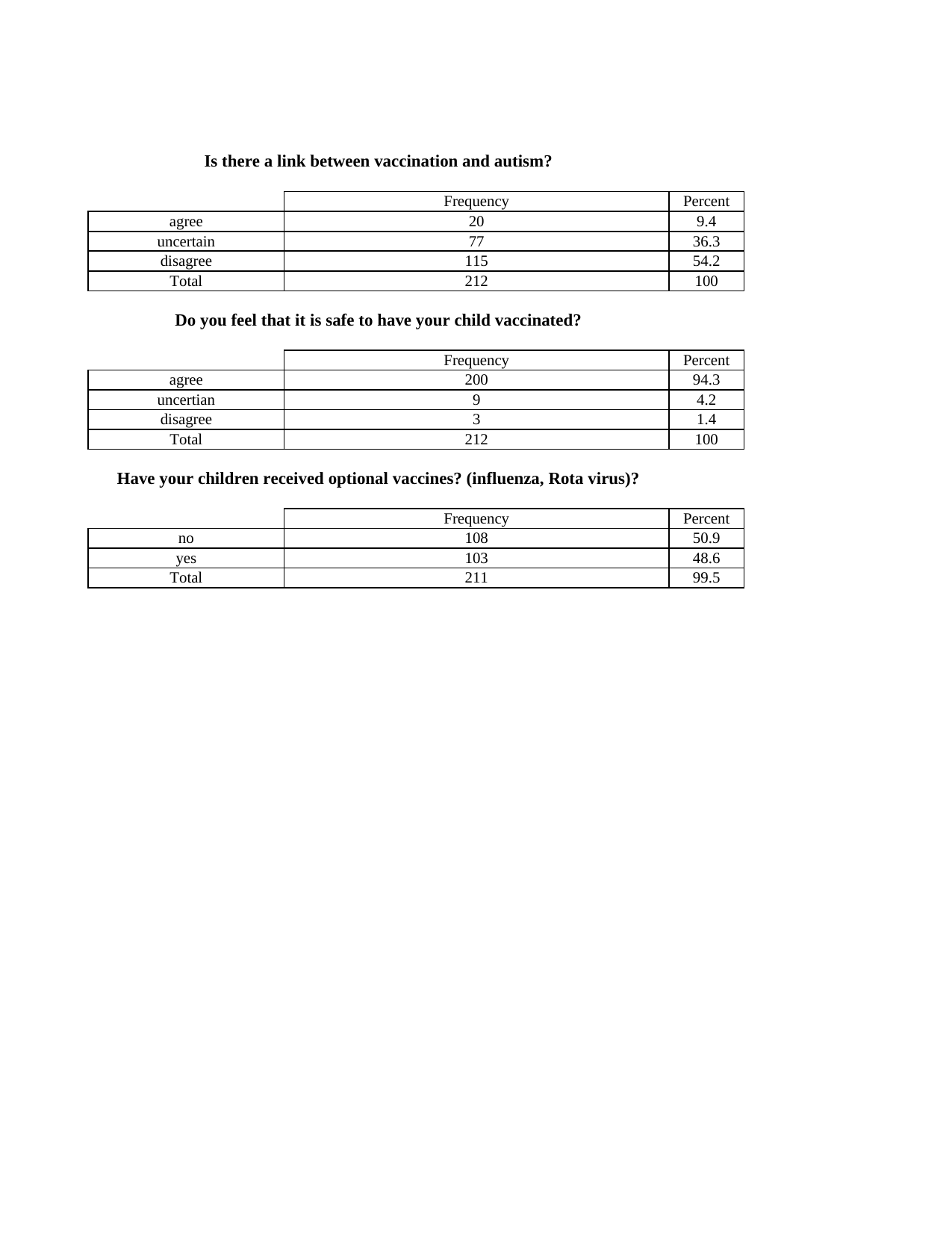

Supplement: S2 Fig — (TIF) [file pone.0322205.s002.tif]
